# Cognition-associated long noncoding RNAs are dysregulated upon severe COVID-19

**DOI:** 10.3389/fimmu.2024.1290523

**Published:** 2024-02-12

**Authors:** Jonathan D. Lee, Isaac H. Solomon, Frank J. Slack, Maria Mavrikaki

**Affiliations:** ^1^ Department of Pathology, Beth Israel Deaconess Medical Center, Harvard Medical School, Boston, MA, United States; ^2^ Department of Pathology, Brigham and Women’s Hospital, Harvard Medical School, Boston, MA, United States; ^3^ Harvard Medical School Initiative for RNA Medicine, Harvard Medical School, Boston, MA, United States

**Keywords:** noncoding RNAs, lncRNAs, cognitive decline, COVID-19, frontal cortex

## Abstract

Severe COVID-19 leads to widespread transcriptomic changes in the human brain, mimicking diminished cognitive performance. As long noncoding RNAs (lncRNAs) play crucial roles in the regulation of gene expression, identification of the lncRNAs differentially expressed upon COVID-19 may nominate key regulatory nodes underpinning cognitive changes. Here we identify hundreds of lncRNAs differentially expressed in the brains of COVID-19 patients relative to uninfected age/sex-matched controls, many of which are associated with decreased cognitive performance and inflammatory cytokine response. Our analyses reveal pervasive transcriptomic changes in lncRNA expression upon severe COVID-19, which may serve as key regulators of neurocognitive changes in the brain.

## Introduction

Neurological symptoms including cognitive decline have been reported in individuals with COVID-19 (1–4). We and others have shown that severe COVID-19 induces widespread changes in protein-coding gene expression in the human frontal cortex (5, 6). However, the brain-related effects of COVID-19 on other RNA species such as long noncoding RNAs (lncRNAs), which may have widespread regulatory roles on transcriptional states despite lacking protein-coding potential (7), remain unclear. LncRNAs, which range from 200 base pairs to hundreds of kilobases, are a relatively understudied class of transcriptional regulators, often acting as scaffolds to recruit transcription factors and effectors to their target genes (8). Their target genes may reside near its gene locus (regulation in *cis*) or across the genome (regulation in *trans*) to regulate transcription (9–11). LncRNAs are expressed at different levels across brain areas and have been linked to synaptic plasticity, memory, and multiple brain disorders (7, 10, 12–17). Due to their potential roles in transcriptional regulation, we sought to determine the breadth of lncRNA changes upon COVID-19.

## Results

**Figure 1:**
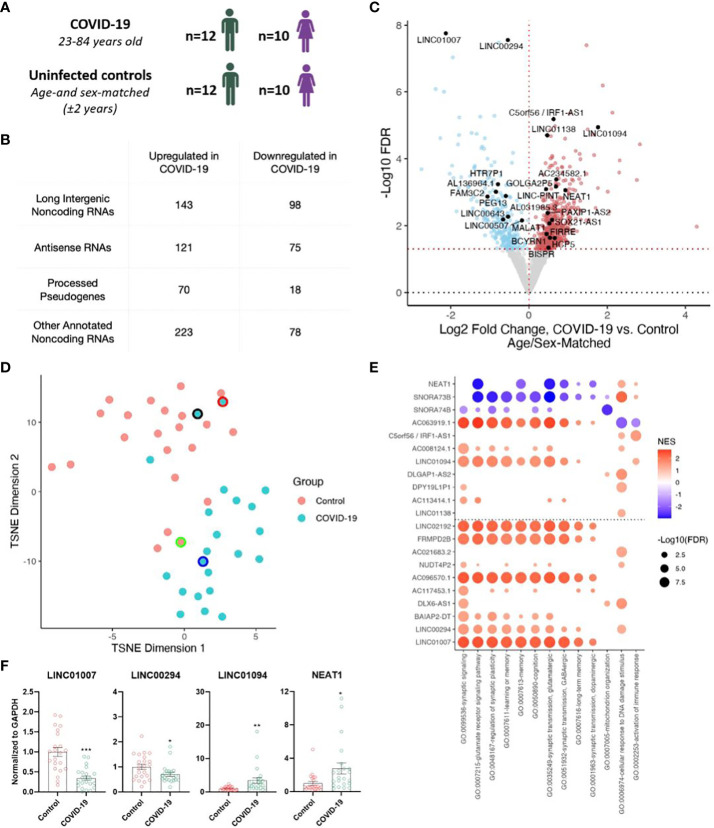
Severe COVID-19 changes the expression of long non-coding RNAs (lncRNAs) in the human frontal cortex. **(A)** Age and sex of individuals with COVID-19 and uninfected age/sex-matched control ( ± 2 years) groups (n=22/group) analyzed in this cohort; for further details see Mavrikaki et al. (5). **(B)** Tabulation of differentially expressed RNA species identified in our sequencing study. **(C)** Volcano plot showing the differentially expressed non-coding genes in the frontal cortex of COVID-19 cases versus age/sex-matched controls (n=22/group). Red points, significantly upregulated genes among COVID-19 cases (false discovery rate/FDR < 0.05). Blue points, significantly downregulated genes among COVID-19 cases. Black points, highlighted significant genes with corresponding gene symbols. **(D)** T-distributed stochastic neighbor embedding (TSNE) analysis of frontal cortex of COVID-19 cases and uninfected age/sex-matched controls, using significant differentially expressed noncoding RNA (ncRNA) expression levels as features. *Black border*, 23-year-old asymptomatic COVID-19 male. *Red border*, 62-year-old COVID-19 female individual with comorbid epilepsy. *Blue border*, 84-year-old COVID-19 female individual with comorbid Alzheimer’s Disease (AD). *Green border*, uninfected age/sex-matched control for the COVID case with comorbid AD. n=22/group. **(E)** Guilt-by-association-based Gene Ontology (GO) biological pathway analysis of top differentially expressed ncRNAs and NEAT1, a lncRNA involved in cognitive processes (18). **(F)** Validation of sequencing data using qRT-PCR. n=22/group. Two tailed unpaired t-test, *p<0.05, **p<0.01, ***p<0.001. LINC01007 t(42)= 5.377, p=0.000003, LINC00294 t(42)= 2.224, p=0.0316, LINC01094 t(42)= 2.844, p=0.0069, NEAT1 t(42)= 2.583, p=0.0134.

We analyzed our previously described total RNA-seq datasets (5), comprising of frontal cortex from 22 individuals with COVID-19 (23-84 years old) and 22 uninfected age- and sex-matched controls (± 2 years) (**Figure 1A**), to annotate both protein-coding and noncoding RNA genes (**Figure 1B**). Differential expression analysis revealed significantly increased (557) and decreased (269) expression levels of noncoding RNAs (ncRNAs) including numerous lncRNAs (long intergenic ncRNAs, antisense RNAs, and processed pseudogenes) associated with COVID-19 (**Figures 1B, C**; **Supplementary Table**). Clustering analysis using transcript abundances of significant differentially-expressed (DE) ncRNAs yielded a separation of COVID-19 cases from controls (**Figure 1D**). Interestingly, the top downregulated lncRNA, LINC01007, and one of the top upregulated lncRNAs LINC01094 upon COVID-19 were previously reported to follow a similar trend as in the brains of aged individuals and Alzheimer’s Disease (AD) patients (19, 20). Additional lncRNAs previously linked to brain aging and AD such as NEAT1, LINC00643, LINC00507, and MALAT1, were also identified (18, 19).

To better understand the roles of the differentially expressed noncoding RNAs in COVID-19, many of which have no known functional roles, we performed guilt-by-association pathway analysis for the top and bottom 10 COVID-19-regulated ncRNAs as well as NEAT1, a well-studied lncRNA involved in brain aging (19) and cognitive function (18). For each ncRNA we ranked the coexpression of protein-coding genes across the transcriptome-profiled samples from The Cancer Genome Atlas (TCGA), spanning multiple tissue samples and genetic backgrounds, and tested for pathway enrichment using these protein-coding gene rankings (21, 22). This analysis implicated many of these lncRNAs in pathways associated with cognitive function (e.g., memory and learning) (23–30) (**Figure 1E**). Finally, we validated the decreased expression of LINC01007 and LINC00294 and increased expression of LINC01094 and NEAT1 by qRT-PCR (**Figure 1F**; **Supplementary Figure 1**). We selected these genes because (1) LINC01007, LINC00294, and LINC01094 are among the top 10 up/down COVID-regulated genes, with LINC01007 and LINC00294 as the two most significantly downregulated lncRNAs, (2) LINC01007 and LINC01094 have been previously associated with aging (19), and (3) NEAT1, also a significantly upregulated lncRNA, is well-established as a regulator of cognitive function (18). Critically, overexpression of NEAT1 impairs cognitive function, whereas knockdown of NEAT1 improves memory in mice (18), in support of a functional role for NEAT1 upregulation in COVID-19-associated cognitive decline.

**Figure 2:**
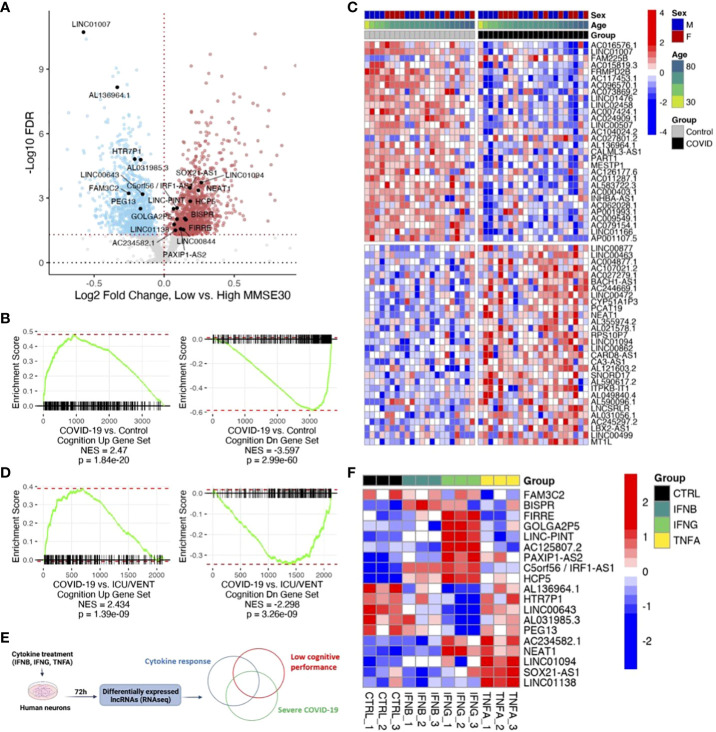
COVID-19 is associated with low cognitive performance-related noncoding RNAs (ncRNAs). **(A)** Volcano plot showing low cognitive performance-related ncRNAs identified in the ROSMAP cohort. Red points, significantly upregulated genes among individuals with low cognitive performance; MMSE scores <25 (false discovery rate/FDR < 0.05). Blue points, significantly downregulated genes with low cognitive performance; MMSE scores <25. Black points, highlighted significant genes with corresponding gene symbols (High MMSE 207 females and 129 males, total 336; Low MMSE 199 females and 98 males, total 297). **(B)** Gene set enrichment analysis (GSEA) of COVID-19-regulated ncRNAs using low cognitive performance-associated ncRNAs as gene sets. DEG ranks were assigned by signed -log10 FDR from frontal cortex transcriptome of COVID-19 versus transcriptome of age/sex-matched control (n=22/group). NES, normalized enrichment score. p, two-tailed GSEA p-value. **(C)** Heatmap of expression values (COVID-19 cohort) of top 30 upregulated ncRNAs and top 30 downregulated ncRNAs overlapping between COVID-19 and low cognitive performance-related ncRNAs. Red represents increased expression; Blue represents decreased expression. **(D)** GSEA of COVID-19-regulated ncRNAs using low cognitive performance-associated ncRNAs as gene sets. DEG ranks were assigned by signed -log10 FDR from frontal cortex transcriptomes of COVID-19 (n=22) versus transcriptomes of uninfected controls with ICU/VENT history (n=9). NES, normalized enrichment score. p, two-tailed GSEA p-value. **(E)** Schematic of *in vitro* cytokine treatment experiment and analytical approach. **(F)** Heatmap of expression values (*in vitro* human neurons) of significant ncRNAs overlapping between COVID-19, cognition, and cytokine response. IFNB: 1ng/ml^-1^; IFNG: 1μg/ml^-1^; TNFA: 100ng/ml^-1^. Red represents increased expression; Blue represents decreased expression.

Next, we sought to evaluate whether the differential expression of these COVID-19-regulated ncRNAs was also associated with poor cognitive performance in humans. We utilized previously published cognitive and transcriptomic data, obtained from the same individuals, in the context of the ROSMAP cohort (31, 32). After splitting those cases (n=633: 406 females and 227 males) by median Mini-Mental State Examination (MMSE) score (high cognitive performance: ≥25, 207 females and 129 males, total 336; low cognitive performance: <25, 199 females and 98 males, total 297) and performing gene expression analysis, we found 1,307 downregulated ncRNAs and 1,322 upregulated ncRNAs in individuals with low cognitive performance (**Figure 2A**; **Supplementary Table**). The larger sample size of the ROSMAP cohort, in comparison to the COVID-19 cohort, likely contributes to increased statistical power and a greater number of significant differentially expressed ncRNAs in the ROSMAP cohort. By Gene Set Enrichment Analysis (GSEA) analysis, we found that ncRNAs associated with severe COVID-19 were also associated with low cognitive performance (**Figures 2B, C**). Moreover, the similarities in ncRNA expression profiles due to COVID-19 and poor cognitive performance are maintained in COVID-19 relative to control cases with history of intensive care unit or ventilator (ICU/VENT) treatment (n=9) (5), in support of potential roles for ncRNAs in COVID-19-induced cognitive changes independent of ICU/VENT-associated treatment (**Figure 2D**).

Finally, as circulating inflammatory factors have been suggested to affect neurological states in COVID-19 (33), we utilized previously published total RNA sequencing data and assessed ncRNA expression changes in primary human neurons upon cytokine treatment (**Figure 2E**; **Supplementary Table**). We found 19 ncRNAs differentially expressed by at least one of IFNB, IFNG, or TNFA that are also differentially expressed in both severe COVID-19 and poor cognition (**Figure 2F**). Of these overlapping genes, LINC01094, NEAT1, and LINC00643 have been previously linked to brain aging and AD (19). Interestingly, loss of NEAT1 not only improves cognitive function (18), but also reduces inflammatory response (34). To obtain further insights into the effects of lncRNAs on protein-coding gene expression, we assessed whether the cognate sense genes (IRF1, PAXIP1, SOX21) of the three significant antisense lncRNAs (C5orf56/IRF1-AS1, PAXIP1-AS2, SOX21-AS1), which often transcriptionally regulate their corresponding sense gene (11), are also significant following cytokine treatment. We found that all three protein-coding genes follow similar expression patterns as the lncRNAs in our *in vitro* neuron datasets (**Supplementary Figure 2**). Of note, IRF1 is also significantly differentially expressed in both COVID-19 and ROSMAP comparisons. This gene is well-implicated in interferon regulation (35–37) and COVID-19 response (37, 38), in support of a role for IRF1-AS1 in the disease. Our analyses highlight the potential for lncRNAs as therapeutic targets to modulate neuroinflammation and mitigate associated cognitive deficits (15).

## Discussion

Given the cognitive decline reported in patients with milder COVID-19 (2), it is tempting to speculate that similar lncRNA expression changes might be found in milder COVID-19 cases. We note, however that our analysis is limited primarily to severe COVID-19 cases due to the availability of relevant specimens. Although we are not statistically powered to make comparisons in milder cases or in asymptomatic individuals with COVID-19, we have included one individual with asymptomatic COVID-19 in our analysis. We found that the ncRNA expression profile from this individual is more representative of control individuals rather than those with severe disease (**Figures 1D**, **2C**).

In summary, we have identified widespread expression changes of numerous lncRNAs in the brain due to severe COVID-19 that are also associated with poor cognition. We link a number of these lncRNAs to transcriptomic changes in neurons upon inflammatory cytokine stimulation. As COVID-19 is associated with cognitive decline (2, 3), our findings suggest key roles for lncRNAs in cognitive decline in individuals with severe COVID-19 and support the idea that inflammation-associated lncRNAs may be targeted to alleviate cognitive deficits observed in COVID-19.

## Materials and methods

### Human biospecimen annotation and RNA-seq library preparation

In this study, we analyzed our previously described total RNA-seq datasets (5). In that cohort, frozen COVID-19 frontal cortex specimens were collected following a protocol for waived consent for the use of excess tissue, approved by the Mass General Brigham Institutional Review Board. Frozen control frontal cortex specimens were obtained from the NIH NeuroBiobank and the NIH HBCC. Clinical features of the COVID-19 cohort have been previously described in Mavrikaki et al. and included 22 cases with pre-mortem or peri-mortem positive testing for SARS-CoV-2 by nasopharyngeal swab qPCR (COVID-19 group) with mean age 61.91 ± 3.1 years (12 males and 10 females), age/sex-matched (± 2 years) uninfected controls without any known psychiatric or neurological disease with mean age 61.86 ± 3.1 years, and an independent group of 9 uninfected cases with history of ICU or ventilator treatment (ICU/VENT) with mean age 57 ± 6.98 years (6 males and 3 females) (5). Total RNA from those samples was extracted using Trizol and phase separation. 450 ng of RNA for the frontal cortex specimens and 80 ng of RNA for the human primary neurons was used for library preparation (5). Libraries were prepared using the KAPA RNA HyperPrep kit with RiboErase (HMR; Roche; #08098131702) following the manufacturer’s recommendations, pooled together (4 runs), and processed for sequencing using NovaSeq 6000 (5). Total RNA-seq data for the COVID-19 cohort and *in vitro* neuron experiment are available at the Gene Expression Omnibus (GEO) with accession number GSE188847.

### RNA-seq alignment and quantification

Raw. fastq sequencing files were aligned to Ensembl v104 using salmon v1.4.0, combining both protein-coding (cdna.all.fa) and noncoding RNA (ncrna.fa) sequences. Annotated gene biotypes were obtained from the Ensembl v96 release (April 2019), as distinction between antisense, processed pseudogene, and long intergenic noncoding RNA were not included in further Ensembl updates. Gene-level abundances were determined using tximport v1.18.0, and differential expression analysis was performed with DESeq2 v1.30.1 using lfcShrink to stabilize variance. Preprocessed ROSMAP gene abundances from n=633 (High MMSE 207 females and 129 males; Low MMSE 199 females and 98 males) and corresponding MMSE cognitive data were obtained from https://www.synapse.org/#!Synapse:syn8691134 and https://www.synapse.org/Portal.html#!Synapse:syn3157322, respectively (39), and differential expression analysis was performed with DESeq2 v1.30.1.

### Pathway analyses

Guilt-by-association pathway analysis of lncRNAs was performed as follows. First, Pearson correlations between the expression levels (log2 transcripts per million + 1) of candidate lncRNAs and those of all protein-coding genes were determined across 9,830 patient transcriptome samples generated as part of The Cancer Genome Atlas Research Network: https://www.cancer.gov/tcga. Protein-coding genes ranked by correlation with each tested lncRNA were used as input for gene set enrichment analysis (fgsea v1.16.0), using gene sets of previously identified Gene Ontology pathways (5). In addition to NEAT1, the top and bottom 10 differentially expressed lncRNAs as ranked by FDR in the COVID-19 cohort and detected in the TCGA dataset were tested for pathway analysis (one snoRNA and one lncRNA were not detected).

Association testing between COVID-19 and ROSMAP cohorts was performed as follows. Signed -log10 FDRs from COVID-19 vs. Control or COVID-19 vs. ICU/VENT comparisons were used to rank ncRNA genes for gene set enrichment analysis via fgsea v1.16.0, filtering out genes with an FDR < 0.5. Cognition-associated gene sets were collated from ROSMAP Poor vs. Normal MMSE comparisons, using significant (FDR < 0.05) ncRNAs. Ensembl gene IDs were used for gene matching in this analysis.

R scripts, reference files, and realigned RNA-seq files used for these analyses are available at https://github.com/jonathandlee12/covid19-brain-lnc. All other reference datasets are either publicly available or will be provided upon reasonable request to the corresponding authors.

### qRT-PCR

A total of 400 ng RNA from each sample was used for cDNA synthesis, and qRT-PCR for orthogonal validation was performed and analyzed as previously described in Mavrikaki et al. (5). GAPDH (Qiagen; QuantiTect primer assay: QT00079247) and RPS18 (Qiagen; QuantiTect primer assay: QT00248682) were used for normalization. Primers for LINC01007 (#qhsaLED0063333), LINC01094 (#qhsaLED0101136), and NEAT1 (#qhsaLED0134812) were purchased from Bio-Rad. Primers for LINC00294 were TGTGTTGTCCTCCAGAATCG (forward) and CCAACCAAGAGCCAACAAAG (reverse) (40), and were synthesized by IDT. Data were analyzed according to the 2^-ΔΔCt^ method (41).

### Transcriptomic data analysis of cytokine-treated neurons

We reanalyzed previously published total RNA-seq data of primary human neurons (ScienCell Research Laboratories, 1520-5) treated with different cytokines (5) which are available on the GEO with accession number GSE188847. Primary neurons were treated with IFN-β (1 ng ml^−1^), IFN-γ (1 µg ml^−1^), TNF (100 ng ml^−1^) or nuclease-free water (control) for 72 h, and RNA was extracted using Trizol/phase separation, and 80ng of RNA was processed for total RNA-seq.

## Data availability statement

The datasets presented in this study can be found in online repositories. The names of the repository/repositories and accession number(s) can be found below: GSE188847 (GEO).

## Ethics statement

The studies involving human specimens were approved by Mass General Brigham Institutional Review Board. The studies were conducted in accordance with the local legislation and institutional requirements. Written informed consent for participation was not required from the participants or the participants’ legal guardians/next of kin in accordance with the national legislation and institutional requirements. Controls were obtained from the NIH Neurobiobank and the NIH HBCC as de-identified samples.

## Author contributions

JL: Data curation, Formal analysis, Investigation, Methodology, Software, Visualization, Writing – original draft, Writing – review & editing. IS: Resources, Writing – review & editing. FS: Funding acquisition, Project administration, Resources, Supervision, Writing – original draft, Writing – review & editing. MM: Conceptualization, Data curation, Formal analysis, Investigation, Methodology, Project administration, Supervision, Writing – original draft, Writing – review & editing.
