# Supplementary material for: Cognition-associated long noncoding RNAs are dysregulated upon severe COVID-19
Source: Front Immunol. 2024 Feb 12;15:1290523. doi: 10.3389/fimmu.2024.1290523 (PMC10894962; doi:10.3389/fimmu.2024.1290523)
Supplement: Supplementary file 1 [file DataSheet_1.pdf]

A

| LncRNA | Control (Ct ± SEM) | COVID-19 (Ct ± SEM) |
|---|---|---|
| **LINC01007** | 27.54 ± 0.368 | 30.81 ± 0.588 |
| **LINC00294** | 27.14 ± 0.385 | 28.86 ± 0.223 |
| **LINC01094** | 29.39 ± 0.185 | 29.26 ± 0.1524 |
| **NEAT1** | 25.59 ± 0.190 | 25.52 ± 0.2287 |

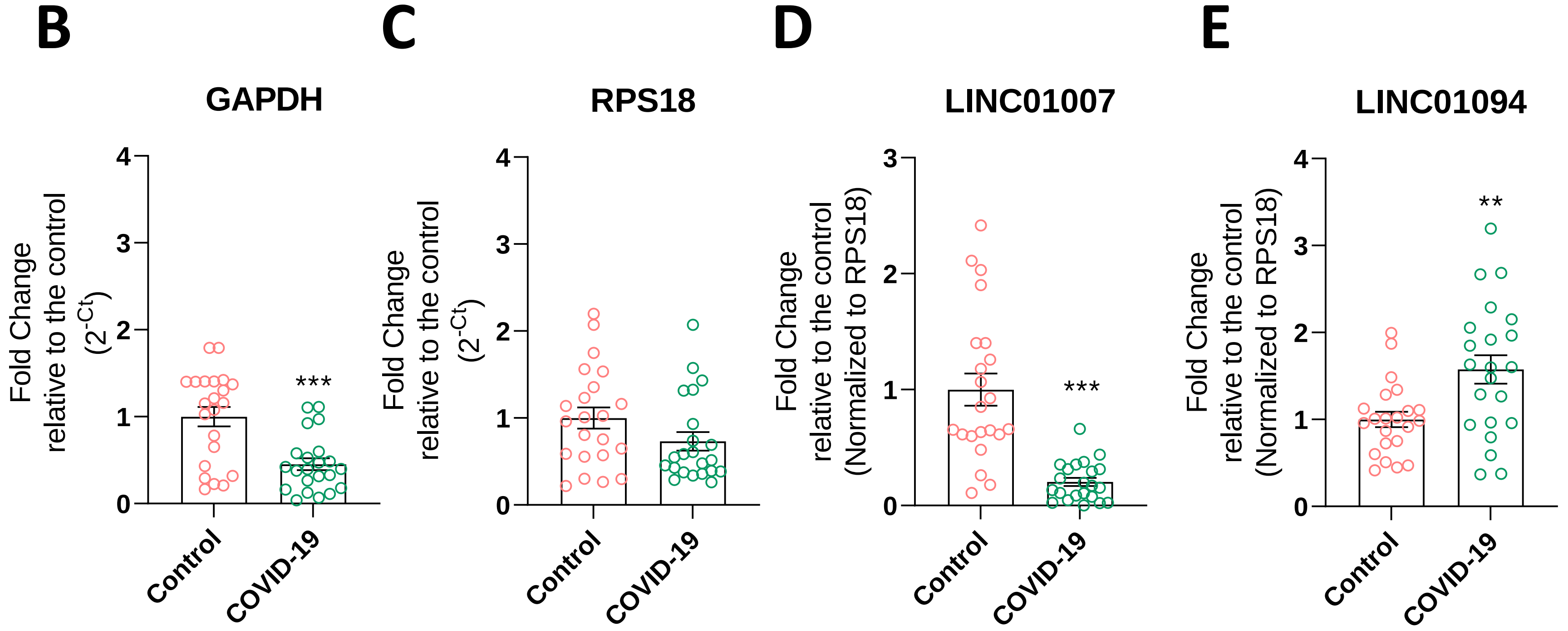


**Supplementary Figure 1.** Validation of sequencing data using qRT-PCR. A. Mean Ct value ± Standard Error of the Mean (SEM) for each lncRNA as assessed by qRT-PCR. B-C. Normalized expression (relative to controls) of GAPDH and RPS18 housekeeping genes (Schmittgen and Livak, 2008). (B) GAPDH $t(42)=4.167$, $p=0.0002$; and (C) RPS18 $t(42)=1.661$, $p=0.1$ (n.s.). D-E. Validation of sequencing data using qRT-PCR with RPS18 for normalization. (D) LINC01007 $t(42)=5.581$, $p=0.000002$; and (E) LINC01094 $t(42)=3.085$, $p=0.0036$; n=22/group. Two tailed unpaired t-test, ***p<0.01, ***p<0.001*.

**A**

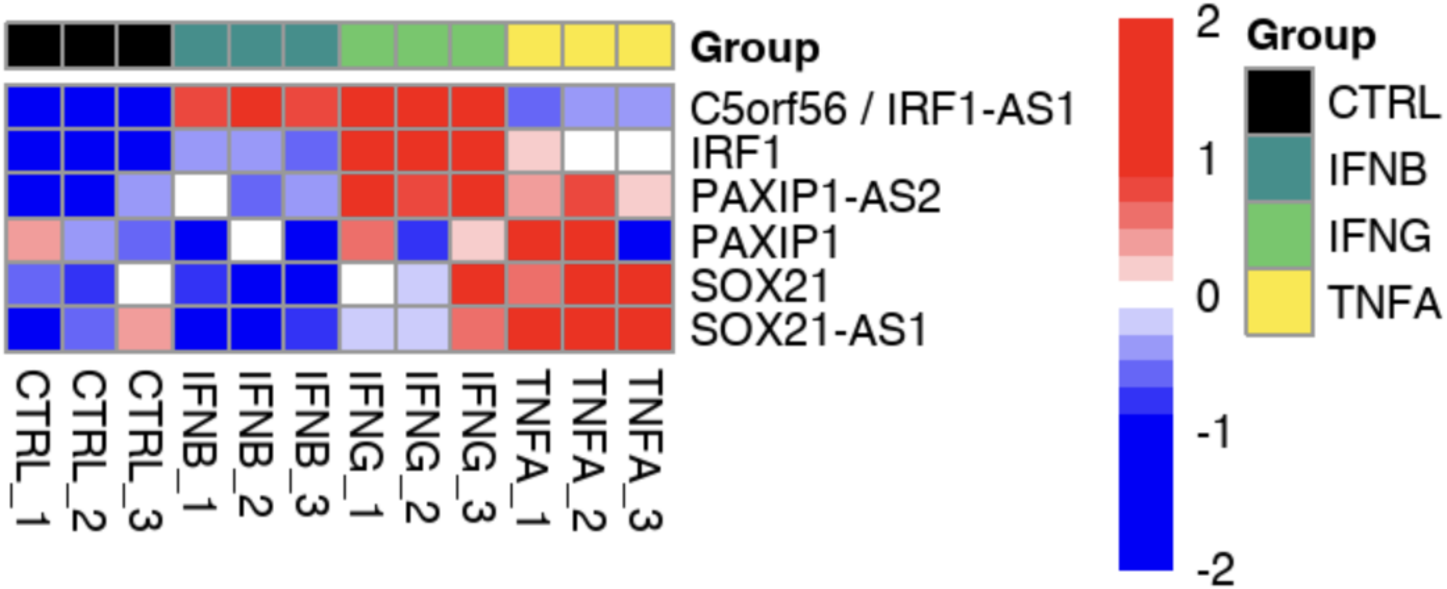


**B**

ROSMAP

| baseMean <dbl> | log2FoldChange <dbl> | lfcSE <dbl> | pvalue <dbl> | padj <dbl> | external_gene_name <chr> |
|---|---|---|---|---|---|
| 98.48484 | 0.121629810 | 0.1580945 | 0.338257438 | 0.521797178 | SOX21 |
| 108.56528 | 0.753534980 | 0.4138491 | 0.001475475 | 0.009727483 | IRF1 |
| 117.42472 | 0.009235717 | 0.1313046 | 0.935794396 | 0.968801481 | PAXIP1 |

COVID

| baseMean <dbl> | log2FoldChange <dbl> | lfcSE <dbl> | pvalue <dbl> | padj <dbl> | external_gene_name <chr> |
|---|---|---|---|---|---|
| 287.0729 | 0.1820605 | 0.05298161 | 8.011126e-05 | 9.882373e-04 | SOX21 |
| 236.7182 | 0.3533188 | 0.07397760 | 8.862296e-08 | 4.991676e-06 | IRF1 |
| 123.0381 | 0.0344032 | 0.02157297 | 9.435370e-02 | 2.207367e-01 | PAXIP1 |

**Supplementary Figure 2.** A. Heatmap of relative expression levels of significant antisense lncRNAs (Figure 2F) and cognate sense protein-coding genes from in vitro cytokine-treated human neurons. IFNB: 1ng/ml$^{-1}$; IFNG: 1µg/ml$^{-1}$; TNFA: 100ng/ml$^{-1}$. B. Differential expression analysis (DESeq2) of cognate sense protein-coding genes as in Supplementary Figure 2A from the ROSMAP High vs. Low MMSE comparison (top) and the COVID-19 vs. Control comparison (bottom).
